# Supplementary material for: From Chemistry to Bioactivity: HS-SPME-GC-MS Profiling and Bacterial Growth Inhibition of Three Different Propolis Samples from Romania, Australia, and Uruguay
Source: Molecules. 2025 Oct 8;30(19):4014. doi: 10.3390/molecules30194014 (PMC12525870; doi:10.3390/molecules30194014)

Supplementary Materials

From Chemistry to Bioactivity: HS-SPME-GC–MS Profiling and Bacterial Growth Inhibition of Three Different Propolis Samples from Romania, Australia, and Uruguay

Radosław Balwierz[1*], Katarzyna Kasperkiewicz[2], Martyna Straszak[2], Daria Siodłak[1], Katarzyna Pokajewicz[1], Ibtissem Ben Hammouda[1], Piotr Paweł Wieczorek[1], Anna Kurek-Górecka[3*], Zenon Czuba[3], and Tomasz Baj[4]

[1] Institute of Chemistry, University of Opole, Oleska 48, 45-052, Opole, Poland
[2] Faculty of Natural Sciences, Institute of Biology, Biotechnology and Environmental Protection, University of Silesia in Katowice, Jagiellonska St. 28, 40-032 Katowice, Poland
[3] Department of Microbiology and Immunology, Faculty of Medical Sciences in Zabrze, Medical University of Silesia in Katowice, Jordana 19, 41-808, Zabrze, Poland
[4] Department of Pharmacognosy with the Medicinal Plants Garden, Medical University of Lublin, 20-001, Lublin, Poland.

Table of Content

Figure S1. Heatmap with hierarchical clustering (HCA) of volatile compounds in propolis samples. Color intensity reflects the z-value, highlighting regional chemical markers. 2

Figure S2. Antimicrobial activity of ethanolic propolis extract from (**01**) Poland, (**02**) Turkey, (**03**) Romania, (**04**) Australia, (**05** and **06**) Uruguay on *Escherichia coli.* **a –** 200 mg/ml concentration of the propolis extract, **b** – 100 mg/ml concentration of the propolis extract, **e** – ethanol 70% 3

Figure S3. Antimicrobial activity of ethanolic propolis extract from (**01**) Poland, (**02**) Turkey, (**03**) Romania, (**04**) Australia, (**05** and **06**) Uruguay on *Staphylococcus aureus.* **a –** 200 mg/ml concentration of the propolis extract, **b** – 100 mg/ml concentration of the propolis extract, **e** – ethanol 70% 4

Figure S4. Antimicrobial activity of ethanolic propolis extract from (**01**) Poland, (**02**) Turkey, (**03**) Romania, (**04**) Australia, (**05** and **06**) Uruguay on *Streptococcus mutans.* **a –** 200 mg/ml concentration of the propolis extract, **b** – 100 mg/ml concentration of the propolis extract, **e** – ethanol 70% 5

Figure S5. Antimicrobial activity of ethanolic propolis extract from (**01**) Poland, (**02**) Turkey, (**03**) Romania, (**04**) Australia, (**05** and **06**) Uruguay on *Yersinia enterocolitica.* **a –** 200 mg/ml concentration of the propolis extract, **b** – 100 mg/ml concentration of the propolis extract, **e** – ethanol 70% 6

Figure S6. Antimicrobial activity of tetracycline (30 μg) on (**A**) *Escherichia coli*, (**B**) *Staphylococcus aureus*, (**C**) *Streptococcus mutans* and (**D**) *Yersinia enterocolitica* 7

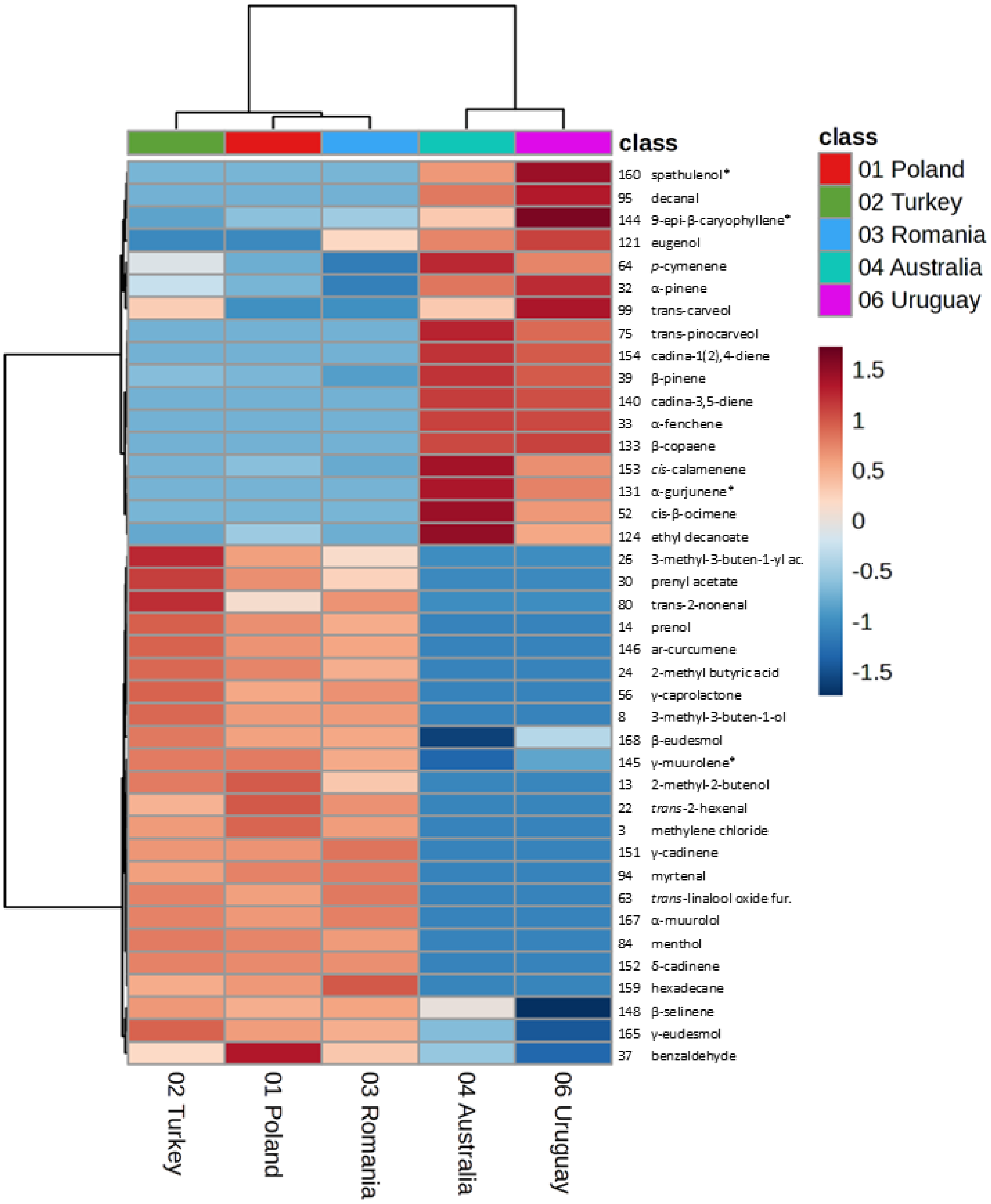

Figure S1. Heatmap with hierarchical clustering (HCA) of volatile compounds in propolis samples. Color intensity reflects the z-value, highlighting regional chemical markers.

Figure S2. Antimicrobial activity of ethanolic propolis extract from (**01**) Poland, (**02**) Turkey, (**03**) Romania, (**04**) Australia, (**05** and **06**) Uruguay on *Escherichia coli.* **a –** 200 mg/ml concentration of the propolis extract, **b** – 100 mg/ml concentration of the propolis extract, **e** – ethanol 70%

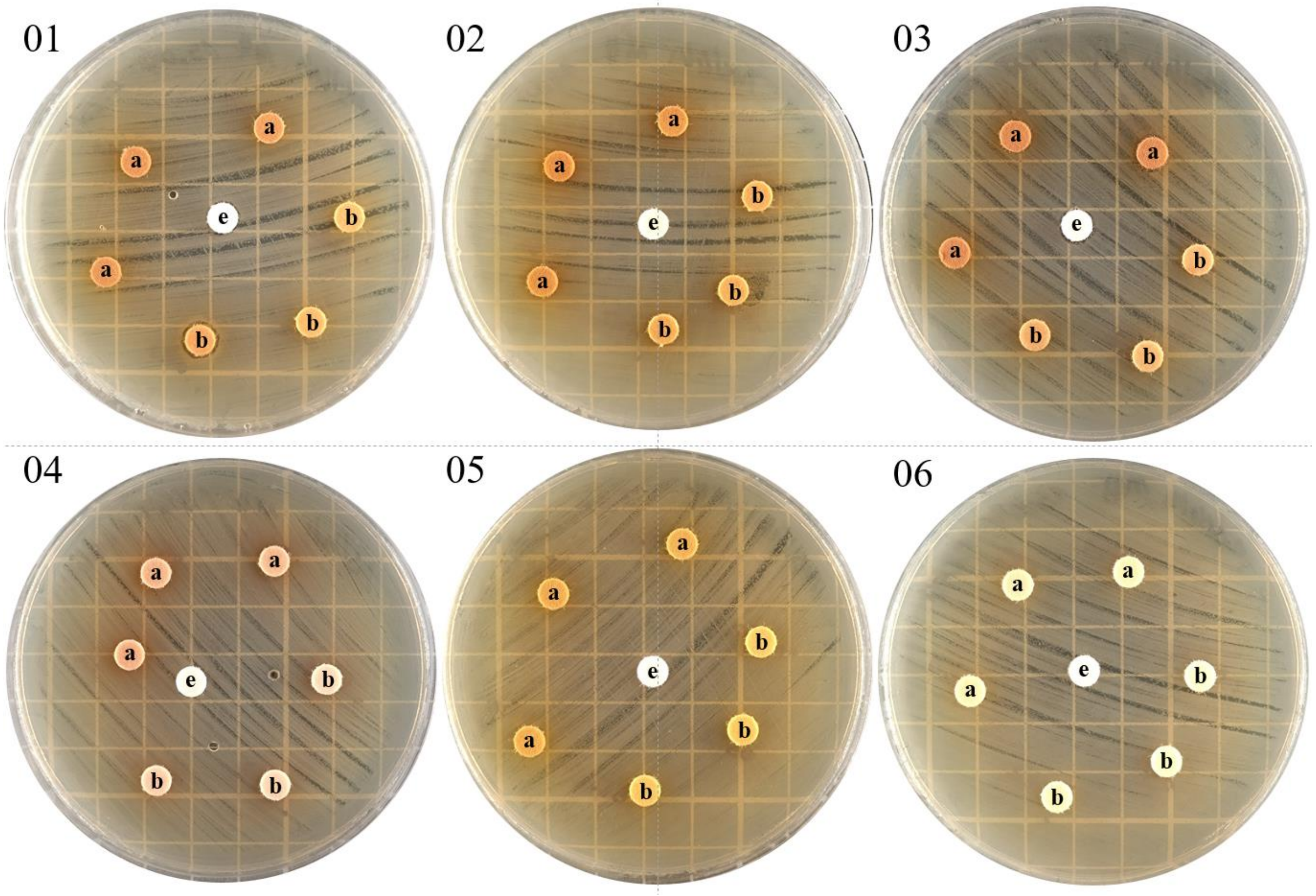

Figure S3. Antimicrobial activity of ethanolic propolis extract from (**01**) Poland, (**02**) Turkey, (**03**) Romania, (**04**) Australia, (**05** and **06**) Uruguay on *Staphylococcus aureus.* **a –** 200 mg/ml concentration of the propolis extract, **b** – 100 mg/ml concentration of the propolis extract, **e** – ethanol 70%

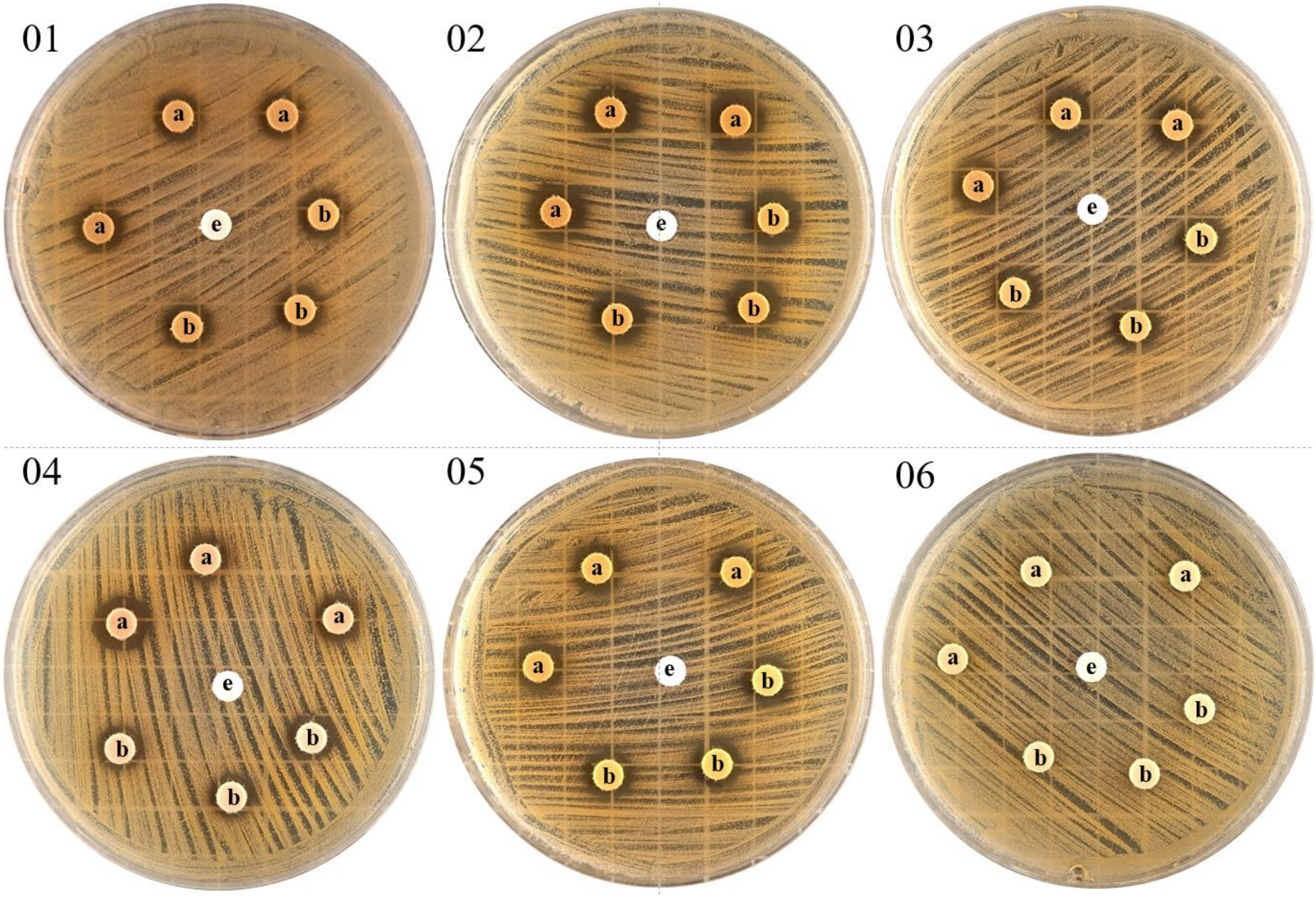

Figure S4. Antimicrobial activity of ethanolic propolis extract from (**01**) Poland, (**02**) Turkey, (**03**) Romania, (**04**) Australia, (**05** and **06**) Uruguay on *Streptococcus mutans.* **a –** 200 mg/ml concentration of the propolis extract, **b** – 100 mg/ml concentration of the propolis extract, **e** – ethanol 70%

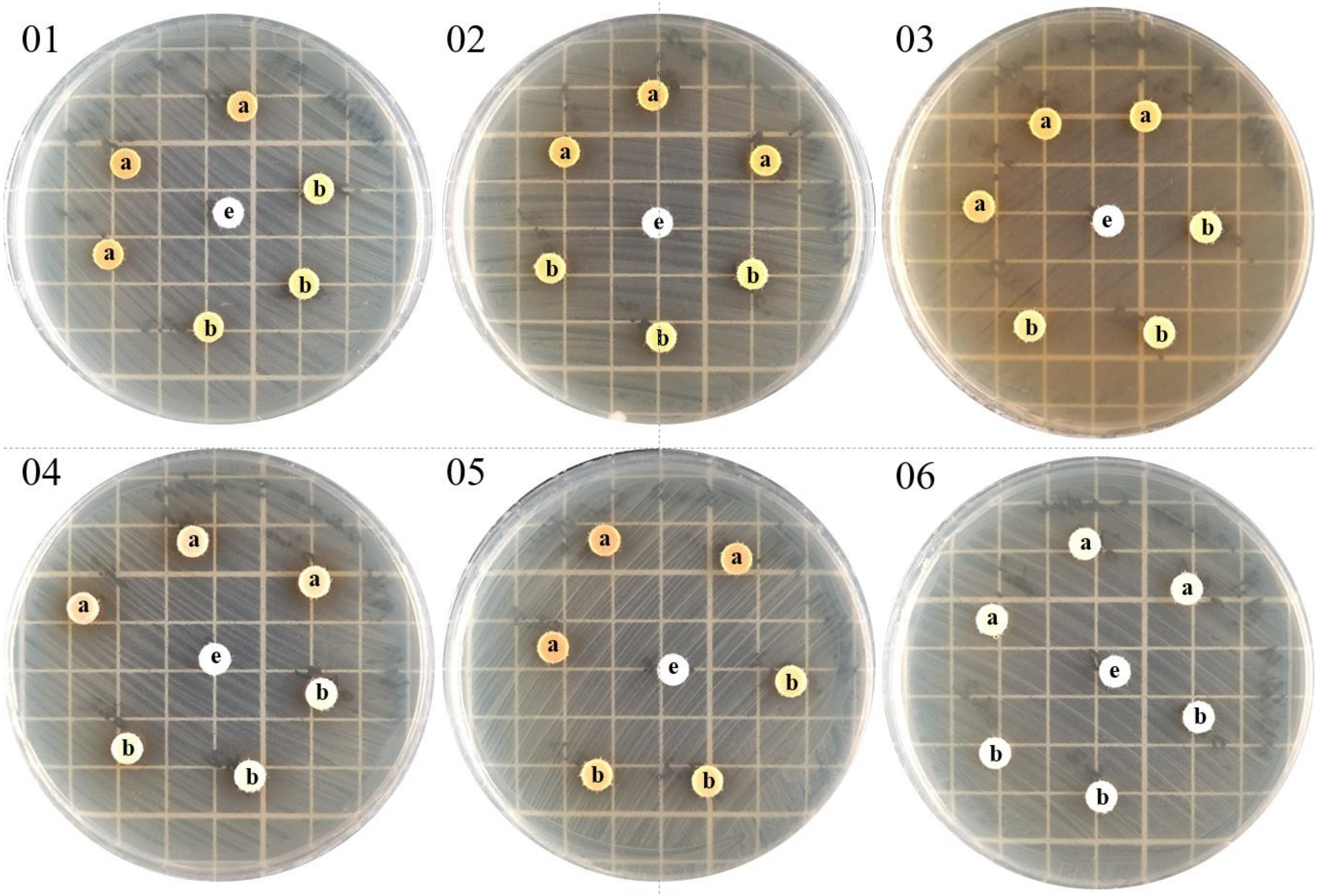

Figure S5. Antimicrobial activity of ethanolic propolis extract from (**01**) Poland, (**02**) Turkey, (**03**) Romania, (**04**) Australia, (**05** and **06**) Uruguay on *Yersinia enterocolitica.* **a** – 200 mg/ml concentration of the propolis extract, **b** – 100 mg/ml concentration of the propolis extract, **e** – ethanol 70%

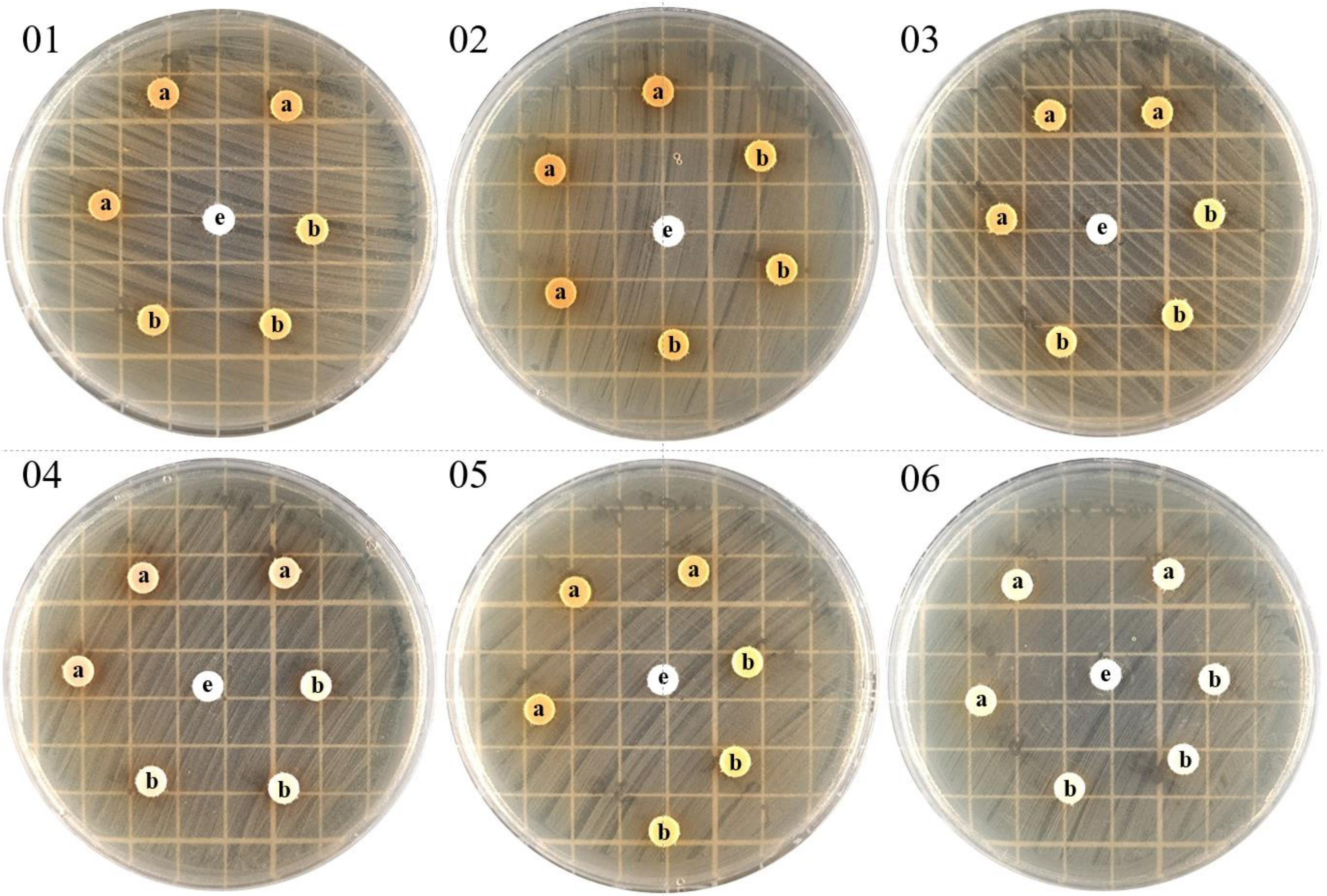

Figure S6. Antimicrobial activity of tetracycline (30 μg) on (**A**) *Escherichia coli*, (**B**) *Staphylococcus aureus*, (**C**) *Streptococcus mutans* and (**D**) *Yersinia enterocolitica*

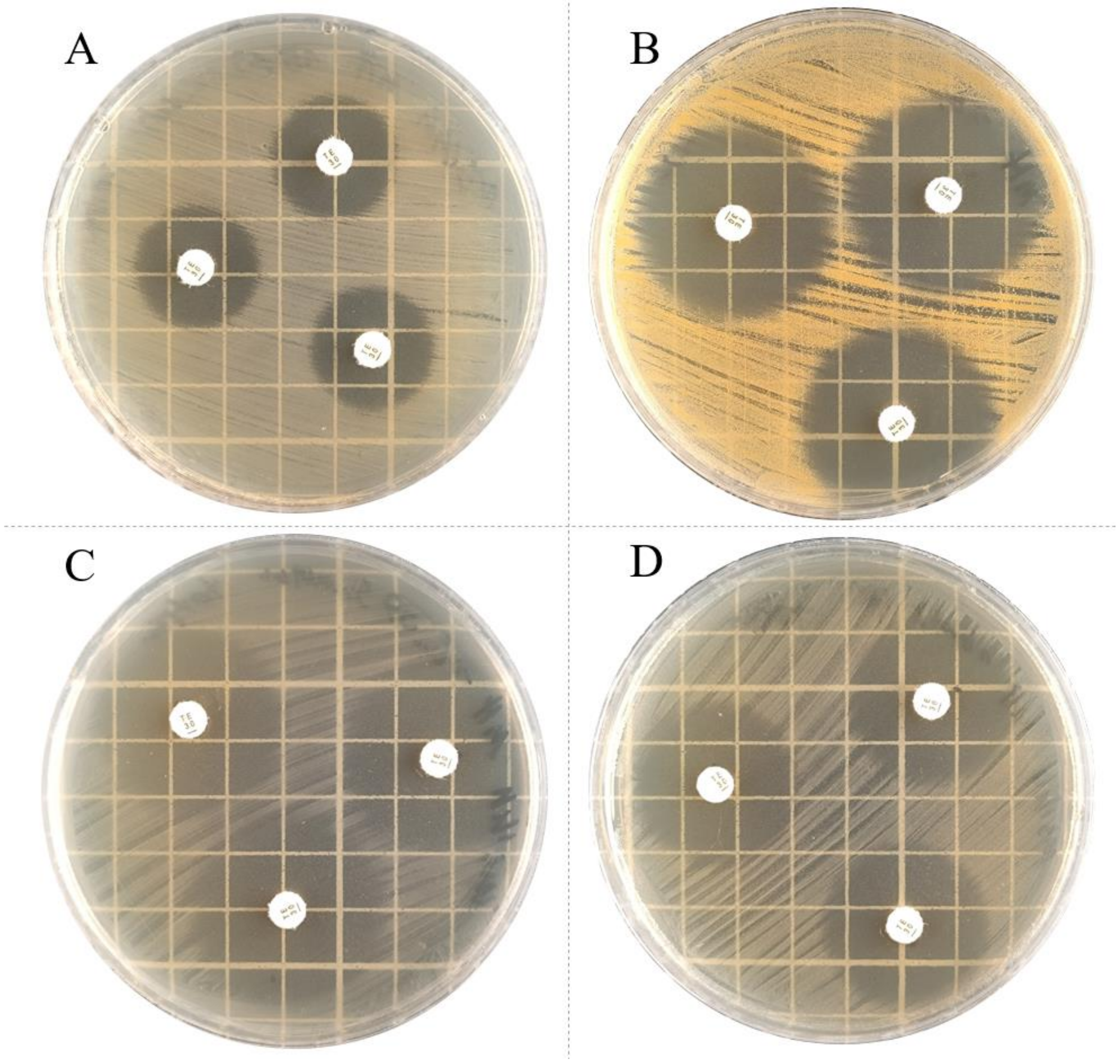

Supplement: Supplementary file 1 [file molecules-30-04014-s001.zip › molecules-3894035-supplementary.pdf]
